# Adoption of routine telemedicine in Norwegian hospitals: progress over 5 years

**DOI:** 10.1186/s12913-016-1743-5

**Published:** 2016-09-20

**Authors:** Paolo Zanaboni, Richard Wootton

**Affiliations:** 1 Norwegian Centre for E-health Research, University Hospital of North Norway, P.O. Box 35, 9038 Tromsø, Norway; 2 Faculty of Health Sciences, The Arctic University of Norway, Langnes, P.O. Box 6050, 9037 Tromsø, Norway

**Keywords:** Telemedicine, Telehealth, Adoption, Implementation, Barriers

## Abstract

**Background:**

Although Norway is well known for its early use of telemedicine to provide services for people in rural and remote areas in the Arctic, little is known about the pace of telemedicine adoption in Norway. The aim of the present study was to explore the statewide implementation of telemedicine in Norwegian hospitals over time, and analyse its adoption and level of use.

**Methods:**

Data on outpatient visits and telemedicine consultations delivered by Norwegian hospitals from 2009 to 2013 were collected from the national health registry. Data were stratified by health region, hospital, year, and clinical specialty.

**Results:**

All four health regions used telemedicine, i.e. there was 100 % adoption at the regional level. The use of routine telemedicine differed between health regions, and telemedicine appeared to be used mostly in the regions of lower centrality and population density, such as Northern Norway. Only Central Norway seemed to be atypical. Twenty-one out of 28 hospitals reported using telemedicine, i.e. there was 75 % adoption at the hospital level. Neurosurgery and rehabilitation were the clinical specialties where telemedicine was used most frequently. Despite the growing trend and the high adoption, the relative use of telemedicine compared to that of outpatient visits was low.

**Conclusions:**

Adoption of telemedicine is Norway was high, with all the health regions and most of the hospitals reporting using telemedicine. The use of telemedicine appeared to increase over the 5-year study period. However, the proportion of telemedicine consultations relative to the number of outpatient visits was low. The use of telemedicine in Norway was low in comparison with that reported in large-scale telemedicine networks in other countries. To facilitate future comparisons, data on adoption and utilisation over time should be reported routinely by statewide or network-based telemedicine services.

## Background

Telemedicine can improve access to healthcare services, especially in sparsely populated and less developed regions, by facilitating contact between patients and providers. Telemedicine has been widely tested over the past 20 years and represents a viable and significant adjunct to the delivery of healthcare [1]. However, adoption into routine practice has been slower than anticipated [2], and evidence for its effectiveness [3, 4] and cost-effectiveness [5] is still limited. Nevertheless, results are improving and several telemedicine applications appear to be promising candidates for widespread use [6]. The widespread deployment of telemedicine might improve quality of life, raise productivity in the health sector [7], avoid travel to underserved populations [8], and contribute to the sustainability of national health systems [9].

**Fig. 1:**
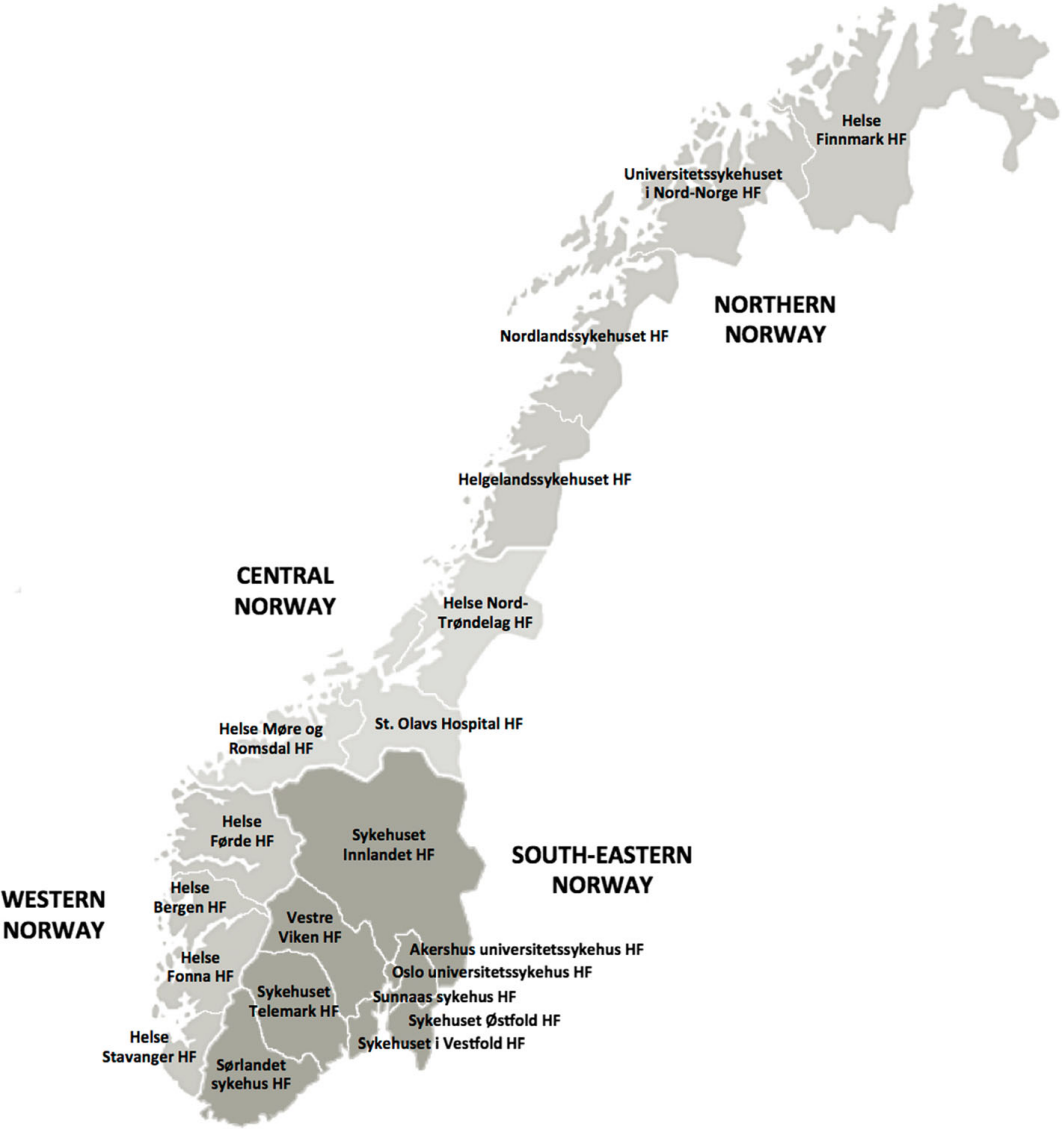
Health regions and public hospitals in Norway

Norway has 5 million inhabitants who are spread over nearly 400,000 square kilometres, making it one of the most sparsely populated countries in Europe [10]. The responsibility for specialist care lies with the state, administered by four Regional Health Authorities (Northern, Central, Western, and South-Eastern Norway). Each region operates a number of public hospitals (Fig. 1). Municipalities are responsible for primary care. Private specialist health facilities are invited as partners to the system on a contractual basis [11]. Despite having one of the highest densities of physicians in Europe, Norway still struggles to ensure geographical and social equity in access to healthcare [12].

Norway is well known for its early adoption of telemedicine to provide services for the population in rural and remote areas in the Arctic [13]. Telemedicine applications have been tested since the early 1990s in the form of pilot projects or small-scale services, some of which have become fully operational [14]. Telemedicine was initially provided as a routine service only to a minor degree, with variations between health regions, but gradually several telemedicine services became ready for large-scale implementation [15]. Recently, adoption of telemedicine was reported in all health regions and most hospitals in Norway. However, the level of use was low [16].

Providing access to telemedicine does not mean that the services will be used to capacity [17]. More efforts are required internationally to provide evidence and data about the deployment of telemedicine [7]. The aim of the present study was to explore the statewide implementation of telemedicine services in Norway over time, to analyse the adoption and level of use of telemedicine by health region, by hospital, and by clinical specialty, and to examine the hypothesis that routine telemedicine is mainly used to increase access to healthcare services in remote areas. A secondary aim was to perform an international comparison of the level of telemedicine activity in Norway with other statewide telemedicine networks.

## Methods

### Data collection

Data on the use of routine telemedicine in Norwegian hospitals were collected from the Norwegian Patient Registry (NPR). The NPR is the central health registry created in 1997 by the Norwegian Directorate of Health to provide data for planning, evaluation, and financing of publicly funded specialised healthcare, as well as for medical and health services research [11]. Data registered in the NPR cover inpatient and outpatient care delivered by publicly funded hospitals. Only telemedicine consultations for which hospitals are reimbursed are included. In Norway, a ‘telemedicine consultation’ is defined as the use of videoconferencing to perform an outpatient consultation, examination, or treatment at a distance. To be registered as a telemedicine activity, a consultation must occur: a) via videoconferencing equipment, meaning that patient and health personnel can see each other through video transmission, b) in real-time, c) between the patient and at least one health professional, of whom at least one is a doctor, from two different physical locations [18]. The use of store-and-forward telemedicine, including the transmission of still images or remote monitoring of a patient’s health parameters, is not covered by any reimbursement scheme in Norway. Contacts occurring by telephone, SMS, or similar means are not considered to be telemedicine consultations. The reimbursement for a telemedicine consultation is equal to that of a traditional outpatient visit.

We sent a formal request to the NPR in April 2014 to obtain data on the telemedicine consultations delivered by Norwegian hospitals from 2009 to 2013. Data related to the outpatient visits were also collected as a means of comparing telemedicine activity with overall hospital activity. The study did not involve human participants, and no personally identifiable data related to individuals were collected. Ethics approvals from the Regional Ethics Committees and informed consents were therefore not required, according to the Norwegian Health Research Act and the Personal Data Act. The Norwegian Directorate of Health approved the request and delivered completed data in November 2014.

### Data analysis

Outpatient visits and telemedicine consultations were stratified by health region, hospital, year, and clinical specialty. Adoption was expressed as the percentage of the number of adopters over the potential users [19]. Since telemedicine can be used to replace outpatient visits, the proportion of telemedicine consultations over the number of outpatient visits was also calculated. The remoteness of each health region was measured through two indexes used to assess the peripherality of Norwegian municipalities: the centrality index (scored 0-20) and the population index (scored 0-10) [20]. Centrality describes the geographic location of a municipality based on the size of the largest urban centre that can be reached within a given travel time. The population index is based on the population density of a municipality. Low values correspond to more isolated and less populated areas, respectively. The indexes for each health region were calculated as the median of the values of all municipalities belonging to that region. Hospitals were arbitrarily stratified by size according to the number of outpatient visits delivered in 2013. Small hospitals had less than 50,000 outpatient visits per year, medium hospitals had 50,000 to 200,000 outpatient visits, while large hospitals had more than 200,000 outpatient visits. Clinical specialties were also arbitrarily stratified by size according to the number of outpatient visits delivered in 2013. Specialties with less than 50,000 outpatient visits per year were considered as low activity, specialties with medium activity had between 50,000 and 200,000 yearly outpatient visits, while high activity was considered as more than 200,000 outpatient visits.

There is a lack of agreed standard measures to calculate telemedicine activity, which makes international comparisons problematic [17]. The number of consultations per site per week has been proposed as a metric to measure telemedicine service use [21]. However, sites may differ in terms of healthcare providers and population served. We selected studies reporting telemedicine activity as the number of telemedicine consultations per year, and compared that to the population served by each telemedicine network. We then calculated the pro capita rate of telemedicine usage.

## Results

### Adoption and use per health region

Table 1 summarises the number of outpatient visits and telemedicine consultations in publicly funded Norwegian hospitals from 2009 to 2013. The number of outpatient visits increased steadily over the 5-year period and in 2013 there were 11.8 % more outpatient visits than in 2009. Growth differed from region to region. The highest growth was recorded in Western Norway and Central Norway, with rates of 16.8 % and 15.8 %, respectively. Growth rates were lower in South-Eastern Norway (9.5 %) and Northern Norway (9.4 %).

**Table 1:** Outpatient visits and telemedicine consultations in the period 2009-2013 in the four health regions in Norway

Health region	Centrality (0-20)^a^	Population (0-10)^a^	Outpatient visits (2009)	Outpatient visits (2010)	Outpatient visits (2011)	Outpatient visits (2012)	Outpatient visits (2013)	Telemedicine consultations (2009)	Telemedicine consultations (2010)	Telemedicine consultations (2011)	Telemedicine consultations (2012)	Telemedicine consultations (2013)
Western Norway	10	0.80	879,911	930,840	947,303	994,769	1,027,463	240 (0.03 %)	246 (0.03 %)	821 (0.09 %)	1586 (0.16 %)	1686 (0.16 %)
Central Norway	11	0.50	695,162	724,617	763,467	784,757	804,753	448 (0.06 %)	23 (0.00 %)	1 (0.00 %)	0 (0.00 %)	32 (0.00 %)
Northern Norway	4	0.20	470,078	484,151	502,839	515,029	514,316	1739 (0.37 %)	876 (0.18 %)	986 (0.20 %)	955 (0.19 %)	991 (0.19 %)
South-Eastern Norway	14	1.30	2,573,532	2,625,076	2,711,593	2,783,087	2,819,054	318 (0.01 %)	41 (0.00 %)	19 (0.00 %)	159 (0.01 %)	170 (0.01 %)
Total	12	0.6	4,618,683	4,764,684	4,925,202	5,077,642	5,165,586	2745 (0.06 %)	1186 (0.02 %)	1827 (0.04 %)	2700 (0.05 %)	2879 (0.06 %)

^a^Values are expressed as median

Values in brackets (%) represent the percentage of telemedicine consultations compared to the number of outpatient visits, by year

**Fig. 2:**
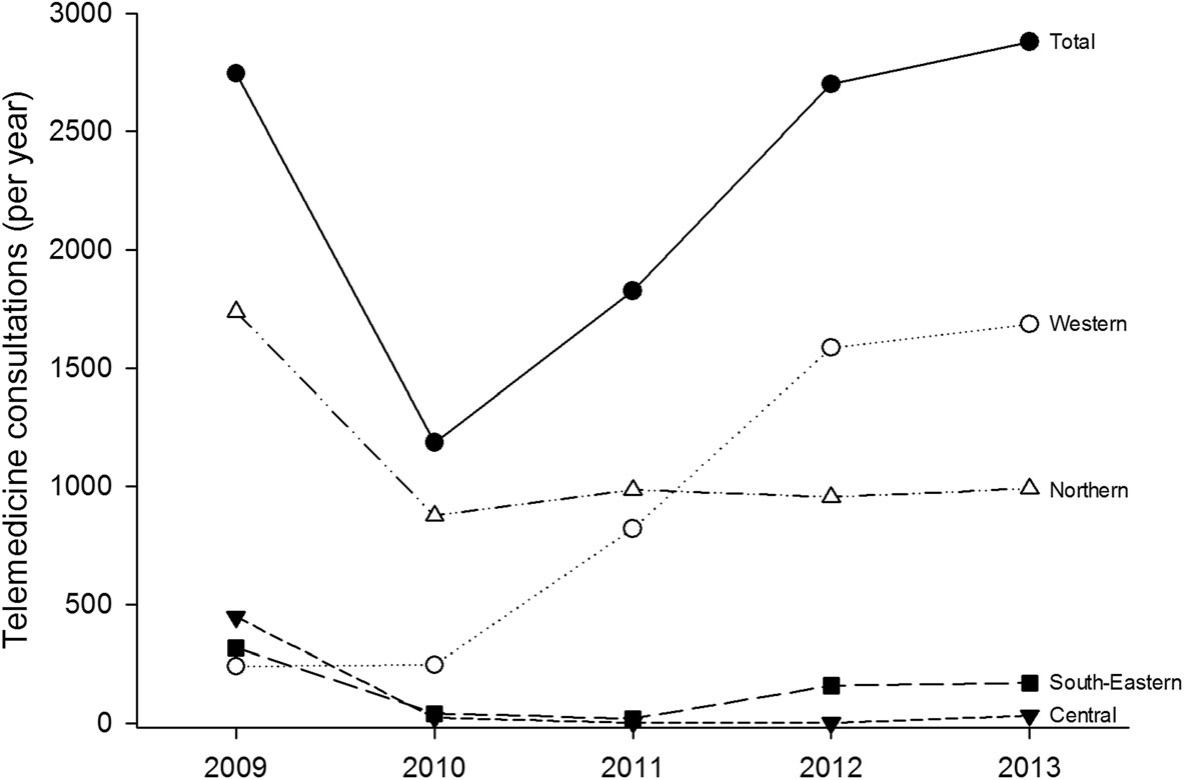
Telemedicine consultations in the period 2009-2013 in Norway and in the four health regions

All four health regions reported the use of telemedicine during the 5-year period, i.e. there was 100 % adoption at the regional level. However, there was a decline in the overall number of telemedicine consultations from 2009 to 2010 (Fig. 2). After 2010 there was a steady increase until 2013. Overall, the number of telemedicine consultations in 2013 was 4.9 % higher than in 2009.

The use of routine telemedicine differed between the health regions (Fig. 2). Western Norway was the only region in which the number of telemedicine consultations increased continually during the study period. In 2013 there were six times more consultations compared to 2009. In the other three regions there was a decline from 2009 to 2010, and then a stable use of routine telemedicine until 2013. Northern Norway, the region delivering most of the telemedicine consultations in 2009, had only half of the consultations in 2010. Western Norway only contributed to the consistent growth characterising the period from 2010 to 2013, thus becoming the region delivering most of the telemedicine consultations.

The use of routine telemedicine seemed to be higher in regions characterised by a lower centrality (Table 1). Similarly, telemedicine appeared to be used to a greater degree in scarcely populated regions. Central Norway seemed to be atypical, using telemedicine less than expected in relation to centrality and population.

### Adoption and use per publicly funded hospital

The number of outpatient visits grew for almost all hospitals over the 5-year period (Table 2). Growth rates ranged from 3.1 % to 63.1 %. Only two hospitals had a change lower than 1 %.

**Table 2:** Outpatient visits and telemedicine consultations in the period 2009-2013 in the publicly funded hospitals in Norway

Hospital	Size	Outpatient visits (2009)	Outpatient visits (2010)	Outpatient visits (2011)	Outpatient visits (2012)	Outpatient visits (2013)	Telemedicine consultations (2009)	Telemedicine consultations (2010)	Telemedicine consultations (2011)	Telemedicine consultations (2012)	Telemedicine consultations (2013)
Western Norway		879,911	930,840	947,303	994,769	1,027,463	240 (0.03 %)	246 (0.03 %)	821 (0.09 %)	1586 (0.16 %)	1686 (0.16 %)
Helse Stavanger HF	Large	236,601	274,315	268,052	279,797	289,860	124 (0.05 %)	201 (0.07 %)	806 (0.30 %)	1583 (0.57 %)	1684 (0.58 %)
Helse Fonna HF	Medium	115,059	118,160	117,049	121,380	122,381	103 (0.09 %)	41 (0.03 %)	13 (0.01 %)	1 (0.00 %)	0 (0.00 %)
Helse Bergen HF	Large	376,996	388,058	409,798	432,519	448,597	0 (0.00 %)	1 (0.00 %)	2 (0.00 %)	2 (0.00 %)	0 (0.00 %)
Helse Førde HF	Medium	110,630	109,995	112,956	114,554	119,052	11 (0.01 %)	2 (0.00 %)	0 (0.00 %)	0 (0.00 %)	2 (0.00 %)
Betanien Hospital (Hordaland)^a^	Small	1675	2059	2097	2104	2192	0 (0.00 %)	0 (0.00 %)	0 (0.00 %)	0 (0.00 %)	0 (0.00 %)
Haugesund San. Revmatismesykehus^a^	Small	21,914	21,066	18,082	24,333	23,915	2 (0.01 %)	0 (0.00 %)	0 (0.00 %)	0 (0.00 %)	0 (0.00 %)
Haraldsplass Diakonale Sykehus^a^	Small	17,036	17,187	19,269	20,082	21,466	0 (0.00 %)	1 (0.01 %)	0 (0.00 %)	0 (0.00 %)	0 (0.00 %)
Central Norway		695,162	724,617	763,467	784,757	804,753	448 (0.06 %)	23 (0.00 %)	1 (0.00 %)	0 (0.00 %)	32 (0.00 %)
St. Olavs Hospital HF	Large	327,390	350,338	368,701	382,669	393,556	448 (0.14 %)	23 (0.01 %)	1 (0.00 %)	0 (0.00 %)	10 (0.00 %)
Helse Nord-Trøndelag HF	Medium	100,797	99,562	109,382	109,110	112,597	0 (0.00 %)	0 (0.00 %)	0 (0.00 %)	0 (0.00 %)	22 (0.02 %)
Helse Møre og Romsdal HF	Large	266,975	274,717	285,384	292,978	298,600	0 (0.00 %)	0 (0.00 %)	0 (0.00 %)	0 (0.00 %)	0 (0.00 %)
Northern Norway		470,078	484,151	502,839	515,029	514,316	1739 (0.37 %)	876 (0.18 %)	986 (0.20 %)	955 (0.19 %)	991 (0.19 %)
Helse Finnmark HF	Medium	55,048	54,132	55,108	59,607	59,092	14 (0.03 %)	33 (0.06 %)	39 (0.07 %)	105 (0.18 %)	76 (0.13 %)
Universitetssykehuset i Nord-Norge HF	Large	214,538	227,831	235,486	238,232	241,248	1325 (0.62 %)	780 (0.34 %)	848 (0.36 %)	558 (0.23 %)	778 (0.32 %)
Nordlandssykehuset HF	Medium	122,723	126,532	130,953	132,566	133,766	147 (0.12 %)	63 (0.05 %)	99 (0.08 %)	292 (0.22 %)	137 (0.10 %)
Helgelandssykehuset HF	Medium	77,769	75,656	81,292	84,624	80,210	253 (0.33 %)	0 (0.00 %)	0 (0.00 %)	0 (0.00 %)	0 (0.00 %)
South-Eastern Norway		2,573,532	2,625,076	2,711,593	2,783,087	2,819,054	318 (0.01 %)	41 (0.00 %)	19 (0.00 %)	159 (0.01 %)	170 (0.01 %)
Sunnaas sykehus HF	Small	2691	3922	3598	3285	4388	0 (0.00 %)	4 (0.10 %)	5 (0.14 %)	132 (4.02 %)	154 (3.51 %)
Vestre Viken HF	Large	287,427	277,960	296,535	306,315	326,293	0 (0.00 %)	3 (0.00 %)	1 (0.00 %)	0 (0.00 %)	1 (0.00 %)
Akershus universitetssykehus HF	Large	175,830	185,536	233,530	254,194	248,798	0 (0.00 %)	0 (0.00 %)	0 (0.00 %)	0 (0.00 %)	0 (0.00 %)
Sykehuset Innlandet HF	Large	317,634	320,325	327,537	335,019	341,459	97 (0.03 %)	14 (0.00 %)	1 (0.00 %)	4 (0.00 %)	1 (0.00 %)
Sykehuset Østfold HF	Large	200,674	195,314	196,563	205,507	212,247	137 (0.07 %)	5 (0.00 %)	2 (0.00 %)	3 (0.00 %)	0 (0.00 %)
Sørlandet sykehus HF	Large	267,781	271,263	279,041	292,567	298,291	74 (0.03 %)	15 (0.01 %)	8 (0.00 %)	18 (0.01 %)	13 (0.00 %)
Sykehuset i Vestfold HF	Large	196,826	195,674	205,989	215,857	213,254	0 (0.00 %)	0 (0.00 %)	2 (0.00 %)	1 (0.00 %)	0 (0.00 %)
Sykehuset Telemark HF	Medium	155,306	164,000	169,598	173,197	154,658	0 (0.00 %)	0 (0.00 %)	0 (0.00 %)	0 (0.00 %)	0 (0.00 %)
Oslo universitetssykehus HF	Large	825,891	859,476	828,164	815,140	832,613	0 (0.00 %)	0 (0.00 %)	0 (0.00 %)	1 (0.00 %)	0 (0.00 %)
Betanien Hospital (Telemark)^a^	Small	14,868	16,983	18,760	19,815	19,642	0 (0.00 %)	0 (0.00 %)	0 (0.00 %)	0 (0.00 %)	1 (0.01 %)
Lovisenberg^a^	Medium	43,071	45,088	52,065	53,489	57,058	10 (0.02 %)	0 (0.00 %)	0 (0.00 %)	0 (0.00 %)	0 (0.00 %)
Martina Hansens hospital^a^	Small	22,934	22,964	25,021	29,528	29,568	0 (0.00 %)	0 (0.00 %)	0 (0.00 %)	0 (0.00 %)	0 (0.00 %)
Revmatismesykehuset Lillehammer^a^	Small	10,701	10,803	12,351	13,916	13,960	0 (0.00 %)	0 (0.00 %)	0 (0.00 %)	0 (0.00 %)	0 (0.00 %)
Diakonhjemmet^a^	Medium	51,898	55,768	62,841	65,258	66,825	0 (0.00 %)	0 (0.00 %)	0 (0.00 %)	0 (0.00 %)	0 (0.00 %)
Total		4,618,683	4,764,684	4,925,202	5,077,642	5,165,586	2745 (0.06 %)	1186 (0.02 %)	1827 (0.04 %)	2700 (0.05 %)	2879 (0.06 %)

^a^Private specialist health facilities

Values in brackets (%) represent the percentage of telemedicine consultations compared to the number of outpatient visits, by year and hospital

**Fig. 3:**
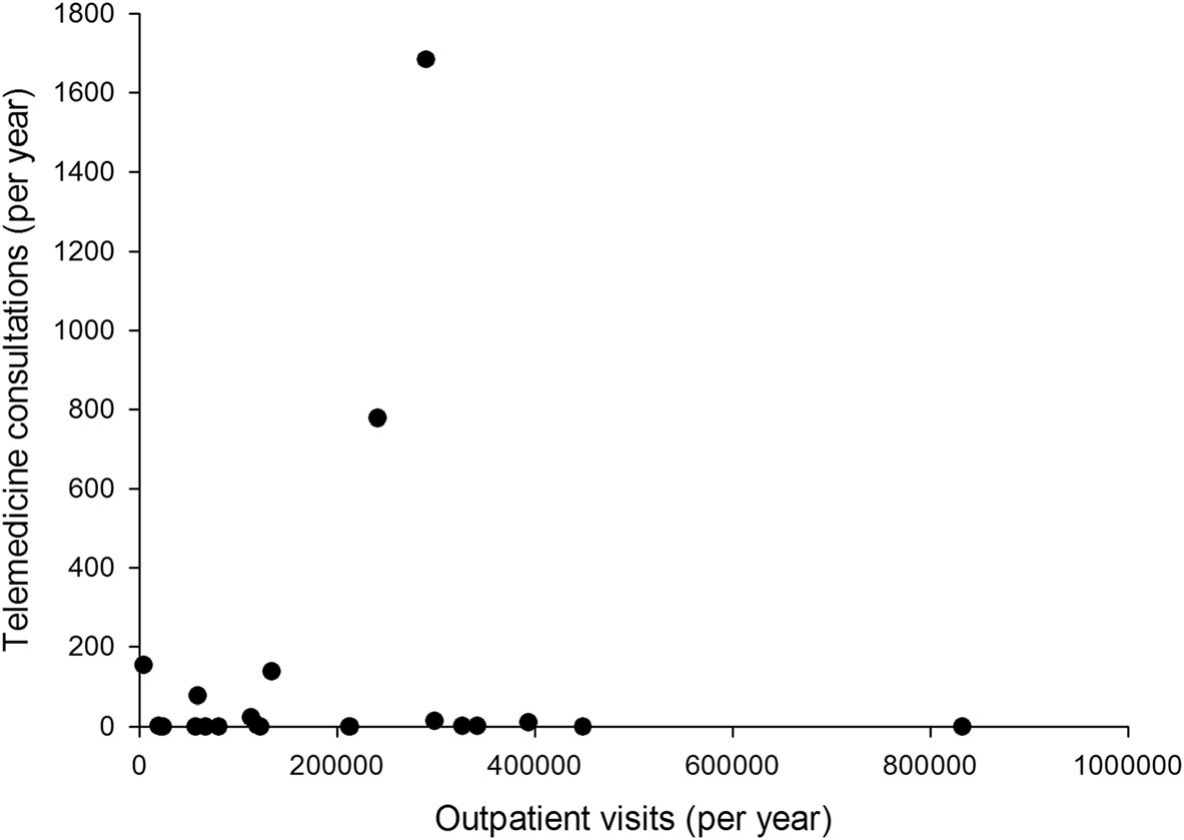
Telemedicine usage compared to hospital size, expressed as outpatient visits in 2013

**Fig. 4:**
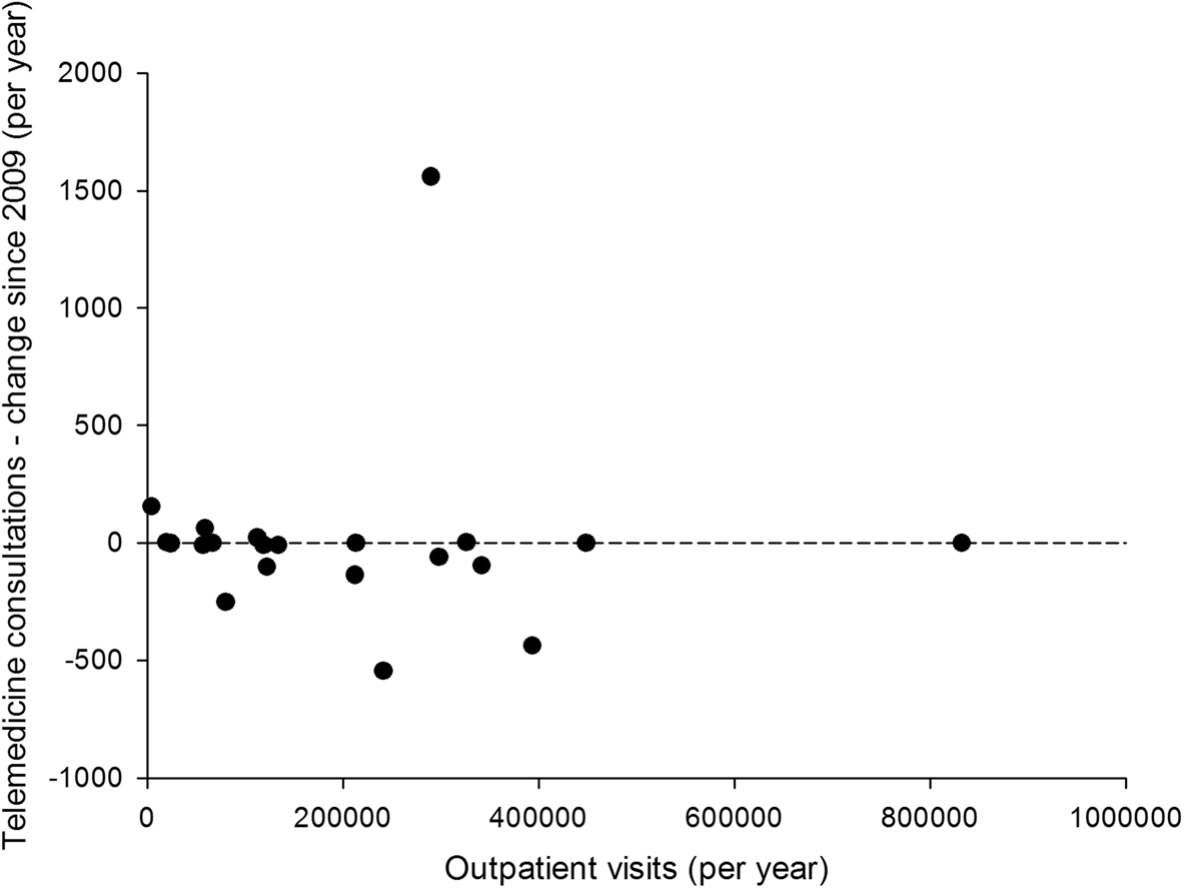
Telemedicine growth compared to hospital size, expressed as outpatient visits in 2013

Twenty-one out of 28 hospitals reported that they had used telemedicine in at least one year during the period 2009-2013, i.e. there was a 75 % adoption at the hospital level. However, not all hospitals used telemedicine continuously over the study period. The number of hospitals reporting telemedicine consultations was 14 in 2010, 15 in 2009 and 2011, and 16 in 2012 and 2013. Telemedicine usage (Fig. 3) and growth (Fig. 4) did not appear to be related to hospital size.

Eleven hospitals delivered more than 50 consultations for at least one year from 2009 to 2013. All the four hospitals in Northern Norway were active in delivering telemedicine over the 5-year study. Three hospitals increased steadily their activity over the period, 7 experienced a decline, while 1 had a variable trend. Helse Stavanger had a large increase of telemedicine activity, with a level in 2013 more than 12 times higher than in 2009, compared to a growth rate of 22.5 % for outpatient visits. This hospital is mainly responsible for the growth trend characterizing Western Norway and the overall national trend as well. Helse Finnmark also increased considerably its telemedicine activity, with a level in 2013 more than 4 times higher than in 2009. This is a medium-sized hospital whose overall outpatient visits grew only by 7.3 % during the same period. Finally, Sunnaas sykehus is a small-sized hospital specialised in rehabilitation. This hospital did not have any telemedicine consultations in 2009, very few in 2010 and 2011, while in 2012 and 2013 the activity was much higher.

Comparing the number of telemedicine consultations to the number of outpatient visits, Sunnaas sykehus was the hospital which performed best, reporting in 2013 a relative use of telemedicine of 3.51 % of all outpatient activity, consisting mainly of rehabilitation visits. That is, the hospital has been replacing outpatient face-to-face visits with remote consultations performed via videoconferencing. Helse Stavanger, the most active hospital delivering telemedicine, reached a relative use of 0.58 % of the overall outpatient activity. Despite this remarkable growth, the level was still low compared to the number of outpatient visits, indicating great potential for using telemedicine to replace traditional outpatient visits. Of the other hospitals, Universitetssykehuset i Nord-Norge had a modest relative use of telemedicine in 2009 (0.61 %) compared to outpatient visits. However, this declined in the following years.

### Adoption and use per clinical specialty

The results show that the use of routine telemedicine differed significantly from region to region, and was only used by some of the Norwegian hospitals. Table 3 shows the overall activity in terms of outpatient visits and telemedicine consultations in the period from 2009 to 2013 stratified by clinical specialty. Data are ordered by relative use of telemedicine compared to the overall outpatient activity in the final year, that is the proportion of telemedicine consultations over the total number of outpatient visits in 2013.

**Table 3:** Outpatient visits and telemedicine consultations in the period 2009-2013 in the different clinical specialties

Clinical specialty	Activity	Outpatient visits (2009)	Outpatient visits (2010)	Outpatient visits (2011)	Outpatient visits (2012)	Outpatient visits (2013)	Telemedicine consultations (2009)	Telemedicine consultations (2010)	Telemedicine consultations (2011)	Telemedicine consultations (2012)	Telemedicine consultations (2013)
Neurosurgery	Small	14,701	16,858	19,144	20,401	21,037	803 (5.46 %)	384 (2.28 %)	469 (2.45 %)	274 (1.34 %)	469 (2.23 %)
Rehabilitation	High	162,434	174,216	194,161	203,567	198,229	389 (0.24 %)	162 (0.09 %)	789 (0.41 %)	1719 (0.84 %)	1853 (0.93 %)
Eye diseases	High	228,680	266,363	287,130	301,316	311,324	154 (0.07 %)	137 (0.05 %)	231 (0.08 %)	229 (0.08 %)	291 (0.09 %)
Endocrinology	Medium	108,866	117,577	119,423	125,502	130,146	16 (0.01 %)	2 (0.00 %)	5 (0.00 %)	23 (0.02 %)	26 (0.02 %)
Cardiovascular diseases	High	222,183	228,739	249,516	260,106	263,619	152 (0.07 %)	58 (0.03 %)	51 (0.02 %)	30 (0.01 %)	42 (0.02 %)
Obstetrics	High	482,661	502,400	513,143	508,829	510,189	102 (0.02 %)	18 (0.00 %)	14 (0.00 %)	87 (0.02 %)	71 (0.01 %)
Digestive diseases	Medium	122,480	142,525	162,385	174,526	175,016	23 (0.02 %)	1 (0.00 %)	11 (0.01 %)	31 (0.02 %)	23 (0.01 %)
Pulmonary diseases	Medium	100,842	112,729	118,963	121,942	124,625	16 (0.02 %)	4 (0.00 %)	20 (0.02 %)	16 (0.01 %)	16 (0.01 %)
Plastic surgery	Medium	52,588	55,257	59,980	56,998	63,413	0 (0.00 %)	1 (0.00 %)	3 (0.01 %)	0 (0.00 %)	7 (0.01 %)
Kidney diseases	Medium	53,312	52,288	54,164	57,058	58,516	12 (0.02 %)	3 (0.01 %)	3 (0.01 %)	11 (0.02 %)	6 (0.01 %)
Urology	Medium	135,833	148,557	160,630	166,324	170,295	49 (0.04 %)	9 (0.01 %)	17 (0.01 %)	32 (0.02 %)	15 (0.01 %)
Neurology	Medium	143,640	150,588	184,850	170,295	170,908	369 (0.26 %)	12 (0.01 %)	20 (0.01 %)	32 (0.02 %)	12 (0.01 %)
General surgery	Medium	166,569	146,575	133,809	128,941	120,954	35 (0.02 %)	10 (0.01 %)	7 (0.01 %)	16 (0.01 %)	4 (0.00 %)
Children’s diseases	High	212,285	215,727	198,207	219,036	218,822	120 (0.06 %)	25 (0.01 %)	5 (0.00 %)	11 (0.01 %)	7 (0.00 %)
Orthopaedic surgery	High	647,839	708,595	739,050	744,014	782,384	60 (0.01 %)	72 (0.01 %)	76 (0.01 %)	139 (0.02 %)	23 (0.00 %)
Oncology and radiotherapy	High	94,416	196,456	206,934	229,263	239,773	71 (0.08 %)	9 (0.00 %)	2 (0.00 %)	6 (0.00 %)	7 (0.00 %)
Skin and venereal diseases	High	197,707	204,552	189,415	222,658	216,227	220 (0.11 %)	239 (0.12 %)	93 (0.05 %)	9 (0.00 %)	5 (0.00 %)
Gastroenterological surgery	Medium	114,129	123,111	126,741	130,506	141,967	9 (0.01 %)	4 (0.00 %)	1 (0.00 %)	25 (0.02 %)	2 (0.00 %)
Anaesthesiology	Small	41,515	44,411	36,567	47,811	47,342	2 (0.00 %)	3 (0.01 %)	1 (0.00 %)	0 (0.00 %)	0 (0.00 %)
Haematology	Medium	61,811	75,488	84,666	91,659	94,146	14 (0.02 %)	0 (0.00 %)	0 (0.00 %)	0 (0.00 %)	0 (0.00 %)
Cardiovascular surgery	Medium	43,410	47,502	53,060	54,176	53,752	1 (0.00 %)	1 (0.00 %)	3 (0.01 %)	4 (0.01 %)	0 (0.00 %)
Ear, nose and throat diseases	High	324,964	333,776	353,326	356,885	365,251	15 (0.00 %)	9 (0.00 %)	3 (0.00 %)	2 (0.00 %)	0 (0.00 %)
General internal medicine	Medium	68,642	65,136	61,923	53,627	55,204	29 (0.04 %)	2 (0.00 %)	1 (0.00 %)	0 (0.00 %)	0 (0.00 %)
Geriatrics	Small	16,338	16,931	18,427	19,370	18,797	10 (0.06 %)	0 (0.00 %)	0 (0.00 %)	2 (0.01 %)	0 (0.00 %)
Infectious diseases	Small	28,603	31,889	34,297	38,322	39,646	1 (0.00 %)	0 (0.00 %)	0 (0.00 %)	1 (0.00 %)	0 (0.00 %)
Maxillofacial and mouth disease	Small	29,634	27,554	26,746	28,005	29,006	10 (0.03 %)	2 (0.01 %)	0 (0.00 %)	0 (0.00 %)	0 (0.00 %)
Other clinical specialities	High	622,864	410,120	367,172	365,899	358,590	61 (0.01 %)	18 (0.00 %)	2 (0.00 %)	0 (0.00 %)	0 (0.00 %)
Pregnancy/parathyroid surgery	Small	2171	9883	15,717	17,186	21,182	0 (0.00 %)	0 (0.00 %)	0 (0.00 %)	0 (0.00 %)	0 (0.00 %)
Rheumatology	Medium	117,566	138,881	155,656	163,420	165,226	2 (0.00 %)	1 (0.00 %)	0 (0.00 %)	1 (0.00 %)	0 (0.00 %)
Total		4,618,683	4,764,684	4,925,202	5,077,642	5,165,586	2745 (0.06 %)	1186 (0.02 %)	1827 (0.04 %)	2700 (0.05 %)	2879 (0.06 %)

Values in brackets (%) represent the percentage of telemedicine consultations compared to the number of outpatient visits, by year and clinical specialty

Neurosurgery and rehabilitation were the clinical specialties where telemedicine was used most, with a relative use in 2013 corresponding to 2.23 % and 0.79 %, respectively. Neurosurgery can be considered as a clinical specialty with a low activity, which appears to be suitable to the use of telemedicine to deliver visits remotely. Early in 2009 over 5 % of all outpatient visits in neurosurgery were delivered via videoconferencing. The use decreased during the following years. Rehabilitation is a clinical specialty with a high level of activity in terms of outpatient visits. Looking at the number of telemedicine consultations in this field, there was a steady growth over the 5 years, and the level in 2013 was almost 4 times higher than in 2009. Rehabilitation became largely the most common clinical specialty in telemedicine. Apart from neurosurgery and rehabilitation, only six other clinical specialties recorded more than 100 telemedicine consultations. These included eye diseases, endocrinology, cardiovascular diseases, neurology, children’s diseases, and skin and venereal diseases. All these specialties, however, experienced a decline in the number of telemedicine consultations occurred from 2009 to 2013.

### International comparison

**Fig. 5:**
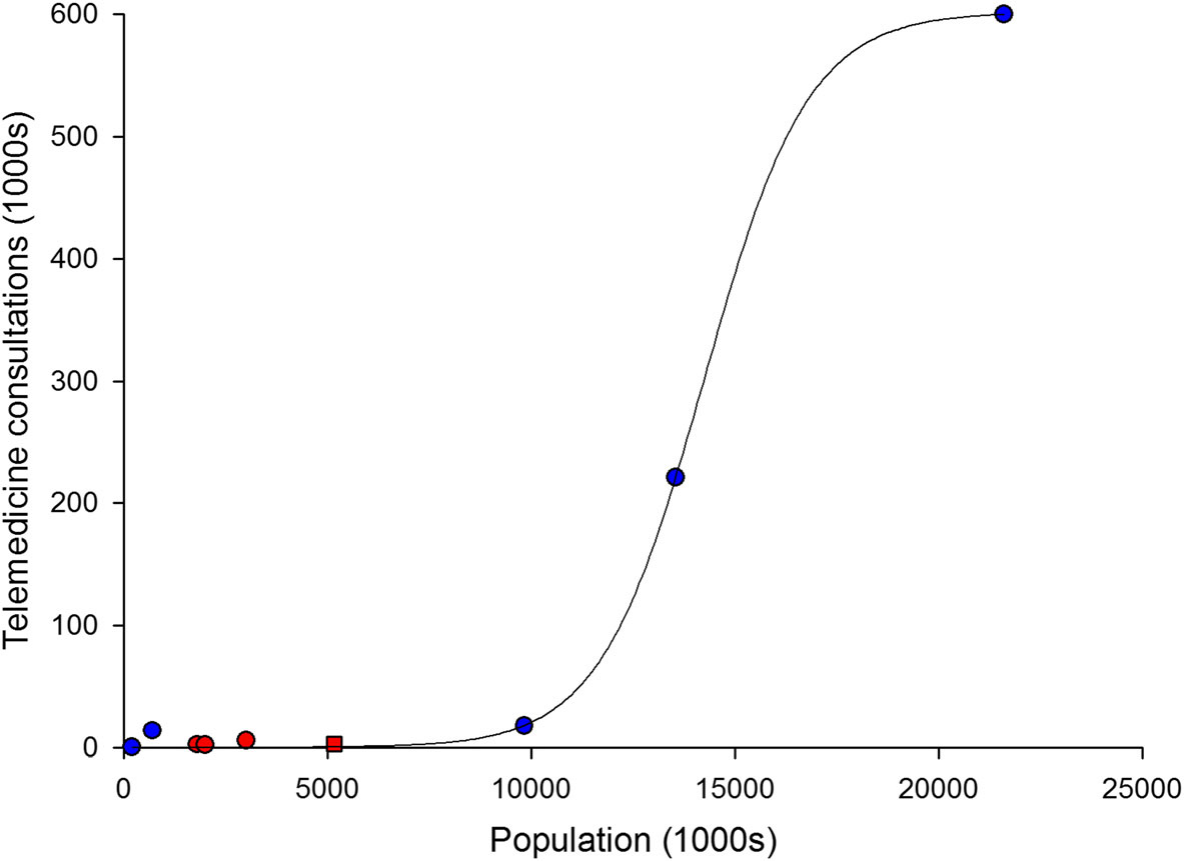
Telemedicine activity in nine statewide networks compared to the population served. The fitted line is a sigmoid. Networks mainly using videoconferencing are shown with red symbols; networks using both videoconferencing and store-and-forward telemedicine are shown with blue symbols. The square symbol represents Norway

Table 4 summarises data from eight different telemedicine networks providing consultations in multiple specialties [17, 22–28] in addition to the data from Norway. The pro capita rate of telemedicine varied from about 1 consultation per year per 1000 persons to over 20 in the largest and well-established telemedicine networks. Figure 5 compares the level of activity in the different statewide networks to the size of the population served by each network. It is apparent that the larger is the population served, the larger is the telemedicine network in terms of sites, and the higher is the telemedicine service usage. This might be explained by the presence of economies of scale. The data can be fitted by a sigmoid curve. While most of the telemedicine networks still have a lower level of activity, the Veterans Health Administration Telehealth Network [22], the Ontario Telemedicine Network [17] and the Alaska Federal Health Care Access Network [26] seem to have succeeded in scaling up both adoption and use of telemedicine. The large telemedicine operations employ both store-and-forward technology and videoconferencing.

**Table 4:** Comparison of telemedicine activity among nine different statewide networks delivering multispecialty services

	Reference	Technology	Year	Network size	Population served	Telemedicine consultations	Pro capita rate^a^
Veterans Health Administration, USA	[Darkins 2014] [22]	VC and SF	2013	152 Medical Centers, 600 community-based outpatient clinics, patients’ homes	21,600,000	600,000	27.8
Alaska, USA	[Kokesh 2011] [26]	VC and SF	2009	248 sites, more than 700 health-care providers	700,000	14,000	20.0
Ontario, Canada	[O’Gorman 2015] [17]	VC and SF	2013	2026 sites	13,550,900	221,353	16.3
African Francophone Telemedicine Network, Bolivia	[Vargas 2014] [23]	VC and SF	2013	more than 20 health institutions	200,000	700	3.5
Alberta, Canada	[Ohinmaa 2006] [24]	VC	2003	212 sites	3,000,000	5766	1.9
Georgia, USA	[Brewer 2011] [25]	VC and SF	2009	51 statewide access points	9,829,211	18,000	1.8
Nebraska, USA	[Meyers 2012] [27]	Mainly VC	2010	over 110 sites	1,800,000	2600	1.4
Western Australia	[Dillon 2005] [28]	VC	2003	104 sites	2,000,000	2151	1.1
Norway	[present study]	VC	2013	28 hospitals	5,165,802	2879	0.6

^a^Pro capita rate: consultations/1000 inhabitants

Abbreviations: *VC* videoconferencing, *SF* store-and-forward

## Discussion

### Overall trend of telemedicine

The present paper reports unique statewide data on the routine use of telemedicine in Norwegian hospitals over a 5-year period. The number of telemedicine consultations increased and followed a similar trend to that of outpatient visits. This presumably reflects the increase of the population and their health needs. An overall growing trend in the delivery of telemedicine has been described in other studies reporting statewide or network-based data over time. Since 1994, telemedicine has become an integral part of the Veterans Health Administration in the United States. Data show a continuous increase in the number of veterans served by telemedicine over 20 years as well as in the number of telemedicine consultations. Telemedicine activity followed an S-shaped innovation curve [22], confirming the hypothesis that telemedicine adoption follows the growth curve typical of health technologies and other innovations [6]. The Ontario Telemedicine Network, the largest telemedicine service provider in Canada and one of the largest in the world, facilitates access to medical care in areas that are often underserved. The number of telemedicine consultations increased in all four Ontario regions from 2008 to 2013, with higher rates in rural North Ontario [17]. The Municipal Department of Health of Belo Horizonte, Brazil, established a telemedicine program in which specialist support was offered to primary care providers. The number of store-and-forward consultations grew from 2006 to 2009 [29]. The African Francophone Telemedicine Network was established to improve access to medical care in the rural Altiplano region of Bolivia, serving a population of about 200,000 inhabitants. The number of telemedicine consultations increased from 2011 to 2013, reaching a yearly average of 700 consultations [23].

### Activity decline in 2010

The data from Norwegian hospitals showed a considerable reduction of telemedicine consultations in 2010. This observed decline might be due to organisational factors [29], such as lack of resources [30], or state-level policies, including reimbursement [31, 32]. In 2009 the Norwegian Health Network was established to provide an infrastructure for secure communication in the health sector in Norway. The implementation and temporary transition to this statewide network might explain the decline of telemedicine in 2010. Another factor to be considered is the nature of the telemedicine consultations. Similar studies showed increased use of store-and-forward consultations over time, while real-time consultations via videoconferencing become less frequent [33, 34]. In 1996, Norway became the first country to implement an official telemedicine fee, without distinction between video and still image solutions [14]. In 2008, however, reimbursement for store-and-forward telemedicine was discontinued, and only telemedicine consultations performed via videoconferencing were reimbursed. While store-and-forward telemedicine appears to be efficient and suitable in routine clinical practice, a lack of reimbursement represents a barrier to its use. We believe that a revision of the current reimbursement policies might create incentives which would result in a wider use of telemedicine by Norwegian hospitals.

### Adoption of telemedicine

Adoption at the regional level was 100 %, that is, use of telemedicine consultations was reported in all four health regions during the period 2009-2013. The results confirm the hypothesis that telemedicine is mainly used to increase access to healthcare services in remote areas with underserved population. Twenty-one out of 28 hospitals reported using telemedicine, i.e. there was 75 % adoption at the hospital level. Thus adoption of telemedicine by Norwegian hospitals appeared to be high, both at regional level and at institutional level. Only a minority of late potential users [19] have still to adopt telemedicine. All the four hospitals in Northern Norway were active in delivering telemedicine over the 5-year study. This might be explained by the higher needs for delivering services remotely due to barriers related to distance and transportation difficulties such as in Northern Norway. Most of the hospitals delivering telemedicine were based in regions characterised by higher remoteness. Adoption rate by percentage of physicians who used the store-and-forward consultations in Belo Horizonte, Brazil, reached 6 % at network level and 18.5 % at district level. Of the adopting physicians, some stopped using telemedicine, while a few remained responsible for most telemedicine consultations [29]. All 21 geographical regions from the Veterans Health Administration Telehealth Network used teledermatology in 2014, with 4 of them collectively reporting 51 % of the patient encounters [34]. The presence of “heavy users” is confirmed by the data from Norway, where only a few hospitals delivered more than 50 telemedicine consultations per year. In the United States, the distribution of telemedicine-related costs covered by Medicare varies across states, services, and specialties. This suggests that factors other than simply rurality or need have driven adoption [35].

### Use of telemedicine

Despite the growing trend and the high adoption, the relative use of telemedicine compared to that of outpatient visits was low. Hospitals in Norway therefore appear not ready yet to replace a substantial proportion of outpatient face-to-face visits with remote consultations. Medicare has been a key payer for telemedicine in the United States since late 1990s, but telemedicine-related costs remain a relatively miniscule part of overall expenditures [35]. A recent report identified six important prerequisites for successful implementation of telemedicine: 1) the national plans exist, but are not well enough coordinated and not supported by sufficient resources; 2) access to a secure communications infrastructure is to a great extent in place; 3) the use of standards is not mandatory; 4) the implementation of Electronic Health Records is very good, but interoperability should be improved; 5) laws should be adapted to the modern way of working; and 6) reimbursement for new ways of health service delivery is not in place [36]. Norway still has some way to go in its use of telemedicine. For example, if it had the same pro capita rate of telemedicine usage as in the Ontario Telemedicine Network [17], the use of routine telemedicine in Norway would increase from 2879 to 78,213 telemedicine consultations every year, almost 30 times more than the current value.

Telemedicine can be used to replace referrals to an outpatient clinic [37], thus reducing travel [8] and unnecessary hospital accesses [38], especially to those living in remote areas. However, it is difficult to estimate the proportion of outpatient visits which could be potentially replaced with telemedicine consultations, since there have been no reports to date of the large-scale use of outpatient telemedicine. It is unlikely that all outpatient visits in all specialties can be replaced by telemedicine visits. On the other hand, there is evidence that in some specialties, substantial numbers of visits can be avoided. Wootton et al. estimated that approximately half of all outpatient visits could be avoided in dermatology [8]. Jaatinen et al. found that a similar proportion of internal medicine and geriatric visits could be avoided in Finland [37]. McGill et al. found that 13 % of visits to a rural fracture clinic in Queensland could be saved by use of telemedicine [39]. If telemedicine was used in just 10 % of all outpatient visits in Norway, this would equate to about 500,000 telemedicine consultations per year, suggesting that there is room for about 100 times as many telemedicine consultations in the future.

## Conclusions

We examined telemedicine adoption in Norway, exploring its level of utilisation overall, by health region, hospital, and clinical specialty. Adoption of telemedicine is Norway is high, with all the health regions and most of the hospitals reporting using telemedicine. The hospitals delivering telemedicine are mostly based in regions characterised by lower centrality and population density. Use of telemedicine has increased over the past five years. However, its relative use compared to the number of outpatient visits is still low. An international comparison shows that only few statewide telemedicine networks seem to have succeeded in scaling up both adoption and use of telemedicine. The present study provides new insights regarding the uptake of routine telemedicine delivered in a large scale. To facilitate future comparisons we recommend reporting data on adoption and utilisation over time from other statewide or network-based telemedicine services.

## Abbreviations

NPR: Norwegian Patient Registry
SF: Store-and-forward
VC: Videoconferencing
